# Pathophysiology of non-cystic fibrosis bronchiectasis in children and adolescents with asthma: A protocol for systematic review and meta-analysis

**DOI:** 10.1371/journal.pone.0294921

**Published:** 2024-04-18

**Authors:** Natali Caroline da Silva, Beatriz Cocato Malagutti, Joelia Maria Costa Dias Ladeira, Milena Baptistella Grotta, Adyleia Aparecida Dalbo Contrera Toro

**Affiliations:** 1 Postgraduate Program in Child and Adolescent Health of the School of Medical Sciences (FCM), State University of Campinas (UNICAMP), Campinas, São Paulo, Brazil; 2 Medical School, Pontifical Catholic University of Campinas, Campinas, São Paulo, Brazil; 3 Center of Integration in Pediatrics (CIPED) of the School of Medical Sciences (FCM), State University of Campinas (UNICAMP), Campinas, São Paulo, Brazil; Bindura University of Science Education, SOUTH AFRICA

## Abstract

**Background:**

The pathophysiological mechanisms by which asthma and bronchiectasis are associated are still unclear. The association of these two diseases can result in more severe symptoms and a greater number of exacerbations.

**Objective:**

The aim of this systematic review is to collect evidence of the pathophysiology of non-cystic fibrosis bronchiectasis with associated asthma in children and adolescents, aged 6–18 years old.

**Methods:**

A systematic and comprehensive search will be performed using eight main databases, PubMed, PubMed PMC, BVS/BIREME, Scopus, EMBASE, Cochrane Library, Scielo and Web of Science. Articles will be searched from the earliest available time to July 2023. The studied population will be composed of children and adolescents with asthma and non-cystic fibrosis bronchiectasis. From the data obtained, all articles found will be transferred to the Rayyan platform. Study selection will follow the Preferred Reporting Items for Systematic Reviews and Meta-Analysis Protocols Checklist (PRISMA P-2015). In addition, if sufficient data are available, a meta-analysis will be conducted. Two independent reviewers will conduct the studies selection, data extraction, and risk of bias assessment. The outcome measures will be to analyze if non-cystic fibrosis bronchiectasis is related to a specific inflammatory profile.

**Discussion:**

A systematic review will provide better knowledge about the etiopathogenesis and causes of the association between asthma and bronchiectasis and its role in the severity and control of asthma. Identifying, selecting and critically evaluating studies on asthma and bronchiectasis, would be possible to illuminate the characteristics of children and adolescents with associated diagnoses and provide information to help individualized treatments in order to control and prevent complications. The findings of this study will be published in a peer-reviewed journal.

**Systematic review registration:**

The systematic review protocol was registered with the International Prospective Register of Systematic Reviews (PROSPERO) in July 2023 (registration number CRD42023440355).

## 1. Introduction

### 1.1. Rationale

The Global Initiative for Asthma (GINA) define asthma as “a heterogeneous disease characterized by chronic airway inflammation,” affecting 1–29% of the population worldwide [1]. In recent years, there has been evidence of an association between asthma and other diseases, including bronchiectasis [2, 3].

Bronchiectasis is a chronic lung disease defined by an irreversible widening of the bronchial tree, usually characterized by a vicious cycle of infection and inflammation, leading to structural damage of the small airways and, eventually, of its surrounding parenchyma [4, 5]. In the literature, bronchiectasis is divided into those caused by cystic fibrosis and those not associated with cystic fibrosis (non-cystic fibrosis bronchiectasis) [6].

The major goal for asthma control, especially in cases of severe asthma, would be identify and manage its comorbidities and that the lack of this identification would be the responsible of therapy failures and for the worsening of symptoms [2, 7, 8].

Asthma would be identified as one of the causes of bronchiectasis, due to its prevalence in the disease, both atopic and non-atopic, in the pediatric population [2, 3]. However, the pathophysiological mechanisms by which the two diseases are associated are still unclear [9].

It is believed that non-cystic fibrosis bronchiectasis in children is caused by repeatedly episodes of external agents associated with genetic vulnerability, however, the other possible etiologies are poorly defined, with heterogeneous risk factors [4, 10]. Diagnosis consists of evaluating the patient’s clinical condition and the results of computed tomography, but it is possible to observe that children with bronchiectasis also develop similar symptoms to asthma, which can be easily confused [4, 11–14].

The association of these two diseases can result in more severe symptoms and a greater number of exacerbations [13, 14], therefore, this systematic review is justified because we believe that by identifying, selecting and critically evaluating studies on asthma and bronchiectasis, would be possible to enlighten the characteristics of children and adolescents with associated diagnoses and provide information to help individualized treatments in order to control and prevent complications.

### 1.2. Objectives

The aim of this systematic review is to collect evidence that supports that non-cystic fibrosis bronchiectasis diagnosed in children and adolescents is associated with control and severity of asthma and worsening lung function. To this end, the proposed systematic review will look to answer the following questions:

- Do children and adolescents with associated asthma and non-cystic fibrosis bronchiectasis show a worse lung function profile evidenced by pulmonary function tests?
- Does the presence of both non-cystic fibrosis bronchiectasis and asthma, in children and adolescents, results in worse asthma control and severity?
- Is non-cystic fibrosis bronchiectasis, in asthmatic children and adolescents, related to a specific inflammatory profile?

## 2. Method

### 2.1. Eligibility criteria

Study selection will follow the Preferred Reporting Items for Systematic Reviews and Meta-Analysis Protocols Checklist (PRISMA P-2015) (S1 Checklist).

#### 2.1.1. Study designs

We will include observational studies (including cohort, case-control and cross-sectional studies). Will be excluded letters to editor, case reports, editorials and narrative reviews. Table 1 shows the inclusion and exclusion criteria that will be used in the study screening, first by title and abstract and then by full text. The study population must be composed of children and/or adolescents with asthma with associated non-cystic fibrosis bronchiectasis.

**Table 1:** Exclusion and Inclusion criteria for study screening.

Inclusion criteria		Exclusion criteria	
**Type of study**	Cohort	**Type of study**	Case reports
	Case-control		Letters to editor
	Cross-sectional		Narrative reviews
			Editorials
**Population**	Children and adolescents with 6–18 years old of all ethnicities and gender diagnosed with asthma and non-cystic fibrosis bronchiectasis^a^	**Population**	Children <6 years old >18 years old with asthma with other causes for bronchiectasis.
**Outcome**	Asthma control	**Outcome**	Outcomes caused by:
	Asthma severity		Cystic Fibrosis
	Pulmonary function		Ciliary dyskinesia
	Inflammation pattern		

^a^confirmed by Computed Tomography (TC)

#### 2.1.2. Participants

We will include children and adolescents aged 6 to 18 years old, diagnosed with asthma with associated non-cystic fibrosis bronchiectasis, irrespective of gender and ethnicity. All participants must be diagnosed with asthma using clearly defined or internationally recognized criteria and have non-cystic fibrosis bronchiectasis confirmed by computed tomography. Participants with cystic fibrosis and ciliary dyskinesia as the cause of bronchiectasis will not be included.

#### 2.1.3. Interventions

Studies to be examined will include any article with children and adolescents with asthma and associated non-cystic fibrosis bronchiectasis. Those articles reporting asthma control, asthma severity, inflammation pattern and pulmonary function will be considered.

#### 2.1.4. Comparators

Given the broad perspective for interventions of interest, comparisons will include the data from groups with no bronchiectasis. It is more likely to appear in experimental studies if they match the inclusion criteria for the review. Comparisons between asthma and non-cystic fibrosis group and asthma without bronchiectasis group may include:

- Asthma severity
- Asthma control
- Pulmonary function
- Inflammation pattern

#### 2.1.5. Outcomes

Outcomes will be collected individually as reported in the studies, including for those data reported as a composite measure. The outcomes must be reported through a validated tool, in accordance with its particularities.

#### 2.1.6. Timing

There will be no restrictions related to timing.

#### 2.1.7. Setting

There will be no restrictions by type of setting.

#### 2.1.8. Language

There will be no restrictions related to language.

### 2.2. Information sources

Literature search strategies will be developed using medical subject headings (MeSH) and text words related to asthma and non-cystic fibrosis bronchiectasis. We will search the following electronic bibliographic databases: PubMed, PubMed PMC, BVS-BIREME, EMBASE, COCHRANE, SCOOPUS, Web of Science and Scielo database with use of boolean operators AND and OR. The search did not utilize a year limit or filters.

### 2.3. Search strategy

This systematic review will use the following search strategy in all databases cited in 2.2: (Child OR Children) OR (Adolescent OR Adolescents OR Adolescence OR Teens OR Teen OR Teenagers OR Teenager OR Youth OR Youths OR "Adolescents, Female" OR "Adolescent, Female" OR "Female Adolescent" OR "Female Adolescents" OR "Adolescents, Male" OR "Adolescent, Male" OR "Male Adolescent" OR "Male Adolescents "") AND (Asthma OR Asthmas OR "Bronchial Asthma" OR "Asthma, Bronchial") AND (Bronchiectasis OR Bronchiectases OR "Saccular Bronchiectasis" OR "Bronchiectasis, Saccular" OR "Saccular Bronchiectases" OR "Cylindrical Bronchiectasis" OR "Bronchiectasis, Cylindrical" OR "Cylindrical Bronchiectases" OR "Varicose Bronchiectasis" OR "Bronchiectasis, Varicose" OR "Varicose Bronchiectases"). (S1 Appendix)

### 2.4. Study records

#### 2.4.1. Data management

The selected literature will be managed using Rayyan, a free web and mobile app for systematic review developed by Qatar Computing Research Institute (QCPI) [15]. Once in Rayyan, duplicate articles will be excluded when identified. Full-text reports will be screen after qualified studies been selected by title and abstract. If necessary, we will provide training to new members of the review team not familiar with the Rayyan platform and the content area prior to the start of the review.

#### 2.4.2. Selection process

The screening will be performed at Rayyan platform, by two reviewers, who will extract the articles independently, first by title and abstract and then by full text. If any disagreement or inconsistency remains, a third investigator will be involved. After reading the full- text reports, it will be decided whether they meet or not the inclusion criteria.

#### 2.4.3. Data collection process

We will extract data independently and in pairs from each eligible study. A calibration exercises before starting the review will be conducted to ensure consistency across reviewers. Data abstracted will include demographic information, methodology, intervention details, and all reported patient-important outcomes. Reviewers will resolve disagreements by discussion. In case of incomplete data, the original author will be contacted and if data cannot be obtained, the study will be excluded.

### 2.5 Data items

The following data will be extracted: study characteristics, type and source of financial support, author names, year of publication, patient characteristics (such as age, gender, symptoms, comorbidities, weight, and height), blood inflammatory biomarkers, pulmonary function tests scores, asthma questionnaires scores, computerized tomography results and other data judge by team as needed for quality assessment and outcomes.

### 2.6. Outcomes and prioritization

The primary outcome:

Non-cystic fibrosis bronchiectasis is related to asthma control. Since severity and control are assessed in asthma by multiple tools, all of them will be considered and evaluated.

The secondary outcomes:

- Non-cystic fibrosis bronchiectasis, in children and adolescents with associated asthma, is related to asthma severity.
- Non-cystic fibrosis bronchiectasis is related to worse pulmonary function in children and adolescents with associated asthma (assessed by spirometry values).
- Children and adolescents with asthma and non-cystic fibrosis bronchiectasis show a specific inflammatory profile. (assessed by IgE, prick test, serum and sputum inflammatory profile or bronchoalveolar lavage).

### 2.7. Risk of bias in individual studies

The risk of bias for analytical cross-sectional studies and prevalence in cross-sectional studies will be assessed using the appropriate tool of the Joanna Briggs Institute (JBI) [16]. For the first one, the tool consists of eight questions that include the possibility of selection bias, measurement bias, and confounding bias.

For prevalence studies, The Critical Assessment Tool for Studies with Prevalence Data is composed of nine questions, which assess from the sample structure of the included study, how it was selected and calculated, to whether the study described in detail how the outcome was evaluated, whether it was evaluated standardized way and with a good response rate, among the study participants included in the prevalence systematic review.

The Newcastle-Ottawa Scale (NOS) [17] will be used for asses risk of bias in case-control and cohort studies, which assesses three study quality parameters: selection, comparability, and exposure assessment. It assigns a maximum score of four for selection, two for comparability, and three for exposure, for a maximum total score of nine. Studies with a total NOS score of five or more are considered to be of moderate to high quality, while those with a total NOS score of less than five are considered low-quality studies.

The quality of evidence for the clinical outcomes will be assessed according to the recommendations of the Grading of Recommendations Assessment, Development, and Evaluation (GRADE) Working Group [18].

### 2.8. Data synthesis

#### 2.8.1. Quantitative synthesis

If studies are sufficiently homogeneous in terms of design and comparator, we will conduct meta-analyses using RevMan 5.4 software. according to the statistical guidelines referenced in the current version of the Cochrane Handbook for Systematic Reviews of Interventions. The Mantel-Haenszel method will be used for the fixed effect model if tests of heterogeneity are not significant. If statistical heterogeneity is observed (I2 > = 50% or P < 0.1) the random effects model will be chosen. If heterogeneity is substantial, we will not perform a meta-analysis; a narrative, qualitative summary will be done.

#### 2.8.2. Measures of treatment effect

- For dichotomous outcomes:

Dichotomous data (asthma control and asthma severity) will be reported as risk ratios (RRs) with 95% confidence intervals (CIs). Statistical significance will be set at P<0.05.

- For continuous outcomes:

Continuous outcomes will be analyzed using weighted mean differences (with 95% CI) or standardized mean differences (95% CI) if different measurement scales are used. Skewed data and non-quantitative data will be presented descriptively.

- Dealing with missing data

When there are missing data, we will attempt to contact the original authors of the study to obtain the relevant missing data. Important numerical data will be carefully evaluated. If missing data cannot be obtained, an imputation method will be used. We will use sensitivity analysis to assess the impact on the overall treatment effects of inclusion of trials which do not report an intention to treat analysis, have high rates of participant attrition, or with other missing data.

- Assessment of heterogeneity

Heterogeneity will be evaluated by the I^2^ test. The value of I^2^ ranges from 0% to 100%. with 0–% to 40% indicating no major heterogeneity, 40–% to 60% indicating moderate heterogeneity, 60–% to 90% indicating substantial heterogeneity, and >90% indicating considerable heterogeneity.

#### 2.8.3 Additional analyses

Subgroup analyses or sensitivity analyses will be used to explore sources of heterogeneity. If the results can be analyzed quantitatively a meta-regression prediction will be performed. Sensitivity analyses will considerer quality components and risk of bias previously appraised by specific tools.

Subgroup analyses will be based on the following:

- Patient characteristic (age, sex, body mass index)
- Step of treatment for asthma according to GINA

#### 2.8.4. Narrative synthesis

If quantitative synthesis is not appropriate, a systematic narrative synthesis will be provided with information presented in the text and tables to summarize and explain the characteristics and findings of the included studies. The narrative synthesis will explore the relationship and findings both within and between the included studies, in line with the guidance from the Centre for Reviews and Dissemination.

### 2.9. Meta-bias(es)

A funnel plot will be used to evaluate publication bias if more than 10 studies are included. RR from each study is plotted against their variance. Asymmetrical appearance of the plot indicates the presence of publication bias. Egger test will be used to test the asymmetry of the funnel plot.

### 2.10. Confidence in cumulative evidence

The quality of evidence will be assessed using the Grading of Recommendations Assessment, Development, and Evaluation (GRADE) system. The evidence will be adjusted to 4 levels: high, moderate, low, or very low.

## 3. Discussion

Both asthma and non-cystic fibrosis bronchiectasis are chronical diseases affecting pediatric population, with great impact in their quality of life, in addition to economic and financial impact to their family and health system [12–14, 19]. Its association, despite being described in literature, requires better comprehension about the pathophysiology involved. We hope that this systematic review will provide better knowledge about the pathophysiology and causes of the association between asthma and bronchiectasis and its role in the severity and control of asthma. It will be possible to open a new range in the understanding of the diseases, which may result in new research and better management of these diseases.

To achieve this goal, it is necessary to carefully evaluate and summarize the evidence published to date.

## Supporting information

S1 Checklist PRISMA checklist / PRISMA-P 2015 checklist. (DOCX)

S1 Appendix Search strategy/Search strategy for PubMed, PubMed PMC, BVS-BIREME, EMBASE, COCHRANE, SCOOPUS, Web of Science and Scielo databases. (DOCX)
