# Supplementary material for: Pathophysiology of non-cystic fibrosis bronchiectasis in children and adolescents with asthma: A protocol for systematic review and meta-analysis
Source: PLoS One. 2024 Apr 18;19(4):e0294921. doi: 10.1371/journal.pone.0294921 (PMC11025797; doi:10.1371/journal.pone.0294921)
Supplement: S1 Appendix — (DOCX) [file pone.0294921.s002.docx]

S1 Appendix - Search Strategy for PubMed, PubMed PMC, BVS-BIREME, EMBASE, COCHRANE, SCOOPUS, Web of Science and Scielo databases.

**Child or Chlidren**

| **MESH** | **Child OR Children** |
| --- | --- |

OR

**Adolescent**

| **MESH** | **Adolescent OR Adolescents OR Adolescence OR Teens OR Teen OR Teenagers OR Teenager OR Youth OR Youths OR "Adolescents, Female" OR "Adolescent, Female" OR "Female Adolescent" OR "Female Adolescents" OR "Adolescents, Male" OR "Adolescent, Male" OR "Male Adolescent" OR "Male Adolescents ""** |
| --- | --- |

AND

**Asthma**

| **MESH** | **Asthma OR Asthmas OR "Bronchial Asthma" OR "Asthma, Bronchial"** |
| --- | --- |

AND

**Bronchiectasis**

| **MESH** | **Bronchiectasis OR Bronchiectases OR "Saccular Bronchiectasis" OR "Bronchiectasis, Saccular" OR "Saccular Bronchiectases" OR "Cylindrical Bronchiectasis" OR "Bronchiectasis, Cylindrical" OR "Cylindrical Bronchiectases" OR "Varicose Bronchiectasis" OR "Bronchiectasis, Varicose" OR "Varicose Bronchiectases"** |
| --- | --- |
